# Di­aqua­bis­(dl-α-lipoato-κ^2^*O*,*O*′)manganese(II)

**DOI:** 10.1107/S2414314625005656

**Published:** 2025-06-27

**Authors:** Farkhod Raxmatovich Jumabaev, Avez Tuymuradovich Sharipov, Vazirakhon Khasanxoja kizi Mannopova, Odil Irgashevich Choriyev, Jamshid Mengnorovich Ashurov

**Affiliations:** a Department of Inorganic, Physical and Colloidal Chemistry, Tashkent Pharmaceutical Institute, 45 Oybek St., Tashkent 100015, Uzbekistan; b Kyungpook National University, Natural Sciences, Department of Pharmacy, Daegu, Democratic People’s Republic of Korea; c Institute of Bioorganic Chemistry, Academy of Sciences of Uzbekistan, 83 M. Ulugbek St., Tashkent 100125, Uzbekistan; Benemérita Universidad Autónoma de Puebla, México

**Keywords:** crystal structure, manganese(II) complex, lipoic acid, hydrogen bond, coordination compound

## Abstract

The manganese(II) coordination compound, [Mn(C_8_H_13_S_2_O_2_)_2_(H_2_O)_2_], with two bidentate α-lipoate ligands and two coordinating water mol­ecules, has been structurally characterized. The cantral Mn^II^ atom lies on a crystallographic twofold rotation axis and adopts a distorted octa­hedral coordination environment, with carboxyl­ate groups chelating the metal in a κ^2^*O*,*O*′-binding mode. One of the sulfur atoms within the 1,2-di­thiol­ane ring exhibits positional disorder over two sites, with refined occupancies of 0.92 and 0.08. The complex is isostructural with previously reported Zn^II^ and Cd^II^ analogues, both of which also display positional disorder in the 1,2-di­thiol­ane ring. The mol­ecules are linked *via* inter­molecular O—H⋯O and C—H⋯S hydrogen bonds into a di-periodic supramolecular framework parallel to (100).

## Abstract 2

The crystal structure of the title di­aqua­bis­(lipoato-κ^2^*O*,*O*′)manganese(II) complex reveals a distorted octa­hedral geometry around the Mn^II^ centre. The supra­molecular framework is consolidated by O—H⋯O and C—H⋯S hydrogen bonds.

## Graphical abstract

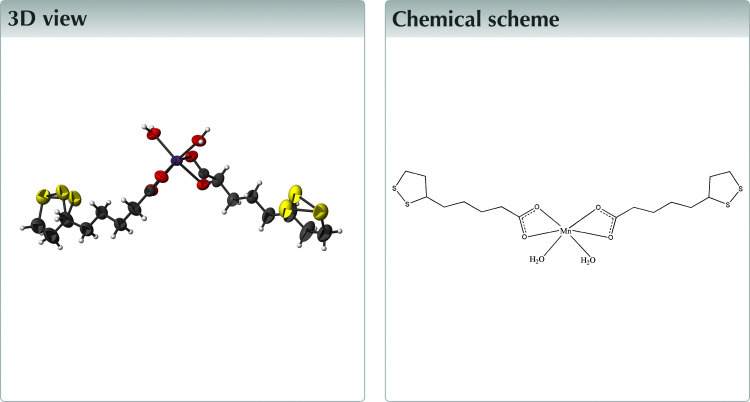

## Structure description

α-Lipoic acid [IUPAC name: 5-(1,2-di­thio­lan-3-yl)penta­noic acid], also known as thio­ctic acid, is a naturally occurring organosulfur compound that acts as a redox-active cofactor in mitochondrial multienzyme complexes such as pyruvate de­hydrogenase and α-ketoglutarate de­hydrogenase (Packer *et al.*, 1995 ▸). As a result of its amphipathic nature, lipoic acid can function across various cellular compartments and participate in redox regulation (Shay *et al.*, 2009 ▸). Its anti­oxidant activity is attributed to its ability to scavenge reactive oxygen species (ROS), regenerate endogenous anti­oxidants, and chelate transition metals (Biewenga *et al.*, 1997 ▸; Solmonson & DeBerardinis, 2018 ▸). These properties make lipoic acid a promising agent for the treatment of oxidative stress-related conditions such as diabetic neuropathy and cardiovascular disorders (Ziegler *et al.*, 2006 ▸; Gorąca *et al.*, 2011 ▸). Importantly, lipoic acid forms stable complexes with metal ions through its di­thiol­ane ring and carb­oxy­lic acid group. These metal complexes, particularly with transition metals, have demonstrated enhanced pharmacological properties including anti­oxidant, anti­cancer, and detoxification activities (Yan *et al.*, 2024 ▸). Chelation with Cu^2+^ and Zn^2+^ has been shown to improve its biomedical applicability, including in nanomedicine and redox modulation. Manganese (Mn), a bioactive transition metal, also exhibits notable therapeutic relevance due to its role in enzymatic activity, immune regulation, and bone formation. Mn-decorated titanium implants and manganese-based nanoparticles have shown osteogenic and immunomodulatory effects, highlighting their potential in tissue engineering and immunotherapy (Wang *et al.*, 2024 ▸; Huang *et al.*, 2023 ▸).

**Figure 1:**
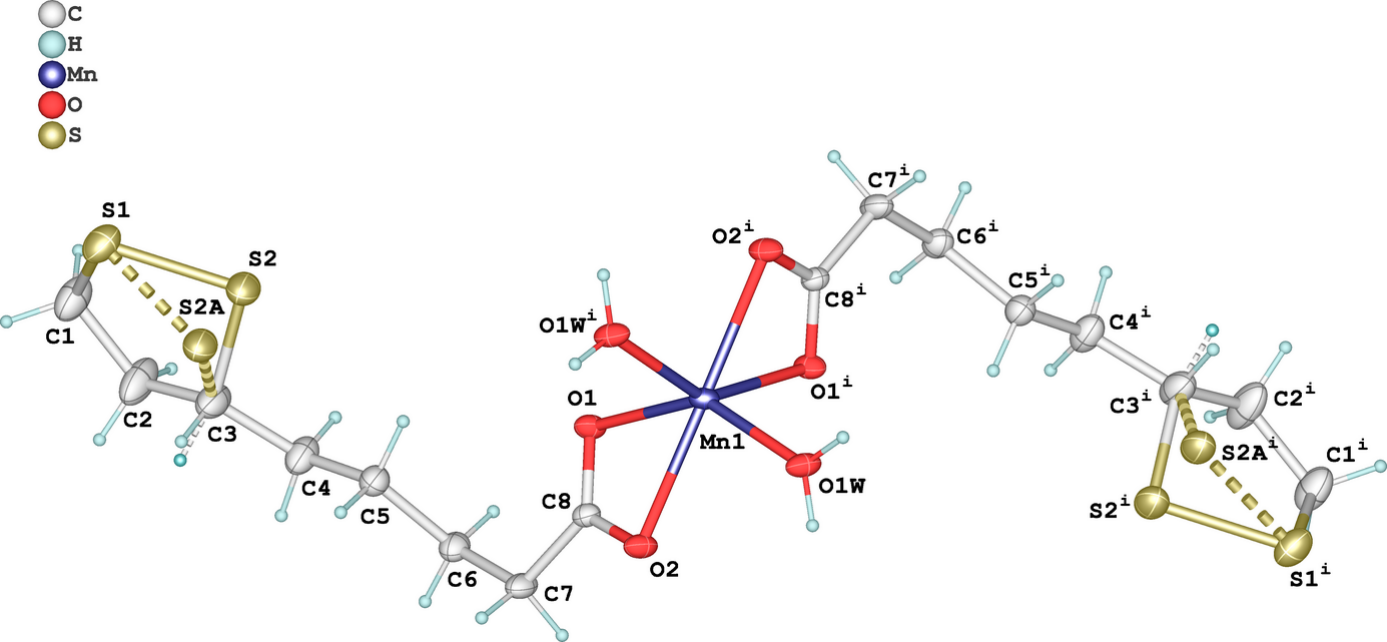
The mol­ecular structure of the [Mn(LIP)_2_(H_2_O)_2_] complex showing the atom-labelling scheme and 50% probability displacement ellipsoids for non-H atoms. Hydrogen atoms are shown as spheres of arbitrary radius. [Symmetry code: (i) 1 − *x*, *y*, 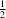 − *z*.]

In this work, we report the synthesis and crystal structure of a novel Mn^II^ complex with dl-α-lipoate (abbreviated LIP). The asymmetric unit of the title compound, [Mn(LIP)_2_(H_2_O)_2_], comprises one half of the mol­ecular unit, with the complete mol­ecule generated by twofold rotation symmetry along the *b*-axis direction, *via* the symmetry operation 1 − *x*, *y*, 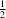 − *z*. The Mn^II^ cation lies on this special position, while all other atoms, including those of the LIP ligands and water mol­ecules, occupy general positions. The central Mn^II^ atom is six-coordinated in a distorted [MnO_6_] octa­hedral shape, defined by four oxygen atoms from two bidentate LIP ligands and two coordinating water mol­ecules (Fig. 1 ▸). The Mn—O bond lengths span from 2.125 (2) (Mn—O1*W*) to 2.258 (2) Å (Mn—O1), with chelate-induced bite angles such as O1—Mn1—O2 = 57.76 (8)°, reflecting notable geometric strain. Notably, the title complex is isostructural with the Cd^II^ complex reported by Strasdeit *et al.* (1997 ▸). In the latter, the Cd—O bond lengths are slightly longer, ranging from 2.226 Å (Cd—O3) for the coordinating water mol­ecule to 2.343 Å (Cd—O2) for the carboxyl­ate oxygen atoms, consistent with the larger ionic radius of Cd^II^ compared to Mn^II^. The S—S bond length in the di­sulfide ring is also similar [2.0443 (18) Å for Mn, 2.047 (3) Å for Cd], indicating structural conservation of the di­thiol­ane moiety across the series. This distortion is further evidenced by the *cis* O—Mn—O bond angles ranging from 87.45 (8) to 108.39 (9)°, and the *trans* angles being reduced to 144.53 (8) and 162.59 (13)°. The coordination environment and geometry are closely comparable to those of the previously reported Zn^II^ analogue, [Zn(LIP)_2_(H_2_O)_2_], in which a similarly distorted octa­hedron is observed (Baumgartner *et al.*, 1996 ▸). The bond lengths in the Mn^II^ complex are slightly elongated, consistent with the larger ionic radius of Mn^II^ relative to Zn^II^. The LIP ligand maintains its five-membered 1,2-di­thiol­ane ring, but displays positional disorder of one sulfur atom. The major component (occupancy 0.92) involves an S1—S2 di­sulfide bridge with a bond length of 2.0443 (18) Å, whereas the minor component (occupancy 0.08) involves an alternative S2*A* position with an S1—S2*A* distance of 2.042 (12) Å. This subtle disorder suggests limited conformational flexibility in the ring, which remains geometrically intact. Similar S—S distances are observed in the Zn^II^ complex [2.025 (4) Å] and in free α-lipoic acid [2.053 (4) Å; Stroud & Carlise, 1972 ▸].

**Figure 2:**
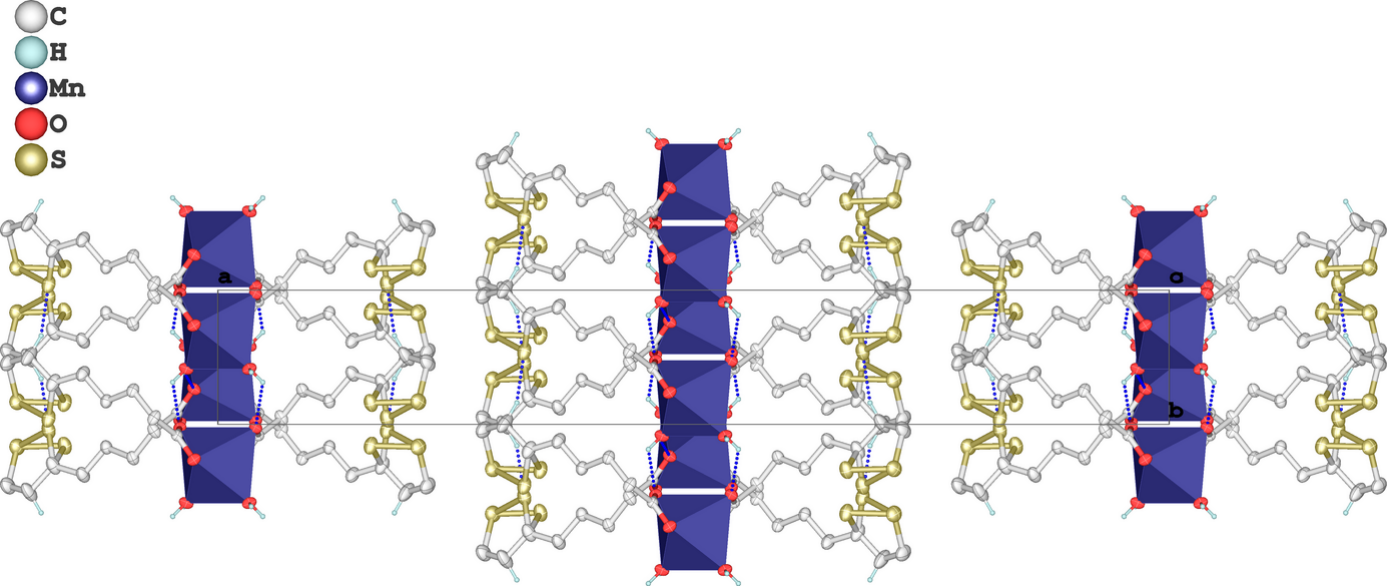
Crystal packing of the [Mn(LIP)_2_(H_2_O)_2_] complex viewed along the *c* axis. Inter­molecular hydrogen bonds are shown as dashed lines. Hydrogen atoms not involved in hydrogen bonding have been omitted for clarity.

The crystal packing is consolidated by a network of classical O—H⋯O hydrogen bonds involving water mol­ecules acting as donors and carboxyl­ate oxygen atoms from adjacent symmetry-related units as acceptors. The O1*W*—H1*W*A⋯O2^i^ and O1*W*—H1*WB*⋯O1^ii^ inter­actions exhibit donor–acceptor distances of 2.725 (3) and 2.718 (3) Å and angles of 159 and 132°, respectively, consistent with the moderately strong hydrogen-bonding geometry typically observed in metal carboxyl­ate systems (Table 1 ▸). In addition, a directional C—H⋯S hydrogen bond between a methyl­ene hydrogen and the minor occupancy sulfur site [C2—H2*B*⋯S2*A*^iii^] is present (Table 1 ▸, last entry), reinforcing the layer cohesion through weak but structurally significant inter­actions. These inter­molecular contacts link the mol­ecules into extended layers parallel to (100), forming a lamellar supra­molecular architecture, as illustrated in Fig. 2 ▸.

## Synthesis and crystallization

To an aqueous solution (2.5 ml) of MnCl_2_·4H_2_O (0.099 g, 0.5 mmol), a sodium salt solution (2.5 ml) of dl-α-lipoic acid (0.206 g, 1 mmol) was added dropwise under constant stirring. The metal-to-ligand molar ratio was 1:2. The resulting mixture was left to stand at room temperature, and pinkish plate-shaped crystals suitable for X-ray diffraction were obtained by slow evaporation over 21 days, yield: 70%. Elemental analysis for C_16_H_30_MnO_6_S_4_ (*M_w_* = 501.58): calculated (%) C, 38.31; H, 6.03; Mn, 10.95; O, 19.14; S, 25.57; found: C, 38.27; H, 5.98; Mn, 10.89; O, 19.12; S, 25.50.

## Refinement

Crystal data, data collection and structure refinement details are summarized in Table 2 ▸. One of the sulfur atoms in the 1,2-di­thiol­ane ring, S2, is disordered over two positions, modelled as S2 and S2*A*, with site occupancies of 0.92 and 0.08, respectively. Geometric and displacement restraints or constraints were applied in the disordered 1,2-di­thiol­ane ring: bonds S1—S2/S2*A* and C3—S2/S2*A* were restrained to have the same distance with a standard deviation of 0.02 Å, and displacement parameters for S2 and S2*A* were constrained to be identical.

## Supplementary Material

Crystal structure: contains datablock(s) I. DOI: 10.1107/S2414314625005656/bh4096sup1.cif

Structure factors: contains datablock(s) I. DOI: 10.1107/S2414314625005656/bh4096Isup2.hkl

CCDC reference: 2466330

Additional supporting information:  crystallographic information; 3D view; checkCIF report

Additional supporting information:  crystallographic information; 3D view; checkCIF report

## Figures and Tables

**Table 1:** Hydrogen-bond geometry (Å, °)

*D*—H⋯*A*	*D*—H	H⋯*A*	*D*⋯*A*	*D*—H⋯*A*
O1*W*—H1*WA*⋯O2^i^	0.85	1.91	2.725 (3)	159
O1*W*—H1*WB*⋯O1^ii^	0.85	2.07	2.718 (3)	132
C2—H2*B*⋯S2*A*^iii^	0.97	2.12	3.043 (18)	158

Symmetry codes: (i) 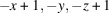; (ii) 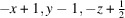; (iii) 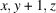.

**Table 2:** Experimental details

Crystal data	
Chemical formula	[Mn(C_8_H_13_O_2_S_2_)_2_(H_2_O)_2_]
*M*_r_	501.58
Crystal system, space group	Monoclinic, *C*2/*c*
Temperature (K)	290
*a*, *b*, *c* (Å)	38.4331 (13), 5.4083 (2), 11.0637 (3)
β (°)	93.566 (3)
*V* (Å^3^)	2295.22 (13)
*Z*	4
Radiation type	Cu *K*α
μ (mm^−1^)	8.32
Crystal size (mm)	0.30 × 0.24 × 0.08

Data collection	
Diffractometer	XtaLAB Synergy, Single source at home/near, HyPix3000
Absorption correction	Multi-scan (*CrysAlis PRO*; Rigaku OD, 2022 ▸)
*T*_min_, *T*_max_	0.419, 1.000
No. of measured, independent and observed [*I* > 2σ(*I*)] reflections	9876, 2214, 1795
*R*_int_	0.052
(sin θ/λ)_max_ (Å^−1^)	0.615

Refinement	
*R*[*F*^2^ > 2σ(*F*^2^)], *wR*(*F*^2^), *S*	0.051, 0.154, 1.05
No. of reflections	2214
No. of parameters	127
No. of restraints	8
H-atom treatment	H-atom parameters constrained
Δρ_max_, Δρ_min_ (e Å^−3^)	0.31, −0.27

Computer programs: *CrysAlis PRO* (Rigaku OD, 2022 ▸), *SHELXT* (Sheldrick, 2015*a* ▸), *SHELXL* (Sheldrick, 2015*b* ▸), *OLEX2* (Dolomanov *et al.*, 2009 ▸) and *publCIF* (Westrip, 2010 ▸).

## full crystallographic data

### Diaquabis[5-(1,2-dithiolan-3-yl)pentanoato-κ2O,O']manganese(II) Crystal data

**Table:**

[Mn(C_8_H_13_O_2_S_2_)_2_(H_2_O)_2_]	*F*(000) = 1052
*M**_r_* = 501.58	*D*_x_ = 1.452 Mg m^−^^3^
Monoclinic, *C*2/*c*	Cu *K*α radiation, λ = 1.54184 Å
*a* = 38.4331 (13) Å	Cell parameters from 4882 reflections
*b* = 5.4083 (2) Å	θ = 4.6–71.2°
*c* = 11.0637 (3) Å	µ = 8.32 mm^−^^1^
β = 93.566 (3)°	*T* = 290 K
*V* = 2295.22 (13) Å^3^	Plate, pinkish
*Z* = 4	0.3 × 0.24 × 0.08 mm

### Diaquabis[5-(1,2-dithiolan-3-yl)pentanoato-κ2O,O']manganese(II) Data collection

**Table:**

XtaLAB Synergy, Single source at home/near, HyPix3000 diffractometer	2214 independent reflections
Radiation source: micro-focus sealed X-ray tube, PhotonJet (Cu) X-ray Source	1795 reflections with *I* > 2σ(*I*)
Mirror monochromator	*R*_int_ = 0.052
Detector resolution: 10.0000 pixels mm^-1^	θ_max_ = 71.4°, θ_min_ = 2.3°
ω scans	*h* = −46→45
Absorption correction: multi-scan (CrysAlisPro; Rigaku OD, 2022)	*k* = −6→6
*T*_min_ = 0.419, *T*_max_ = 1.000	*l* = −13→12
9876 measured reflections	

### Diaquabis[5-(1,2-dithiolan-3-yl)pentanoato-κ2O,O']manganese(II) Refinement

**Table:**

Refinement on *F*^2^	Primary atom site location: dual
Least-squares matrix: full	Secondary atom site location: difference Fourier map
*R*[*F*^2^ > 2σ(*F*^2^)] = 0.051	Hydrogen site location: mixed
*wR*(*F*^2^) = 0.154	H-atom parameters constrained
*S* = 1.05	*w* = 1/[σ^2^(*F*_o_^2^) + (0.0936*P*)^2^ + 1.0204*P*] where *P* = (*F*_o_^2^ + 2*F*_c_^2^)/3
2214 reflections	(Δ/σ)_max_ = 0.005
127 parameters	Δρ_max_ = 0.31 e Å^−^^3^
8 restraints	Δρ_min_ = −0.26 e Å^−^^3^

### Diaquabis[5-(1,2-dithiolan-3-yl)pentanoato-κ2O,O']manganese(II) Fractional atomic coordinates and isotropic or equivalent isotropic displacement parameters (Å^2^)

**Table:**

	*x*	*y*	*z*	*U*_iso_*/*U*_eq_	Occ. (<1)
Mn1	0.500000	0.17395 (11)	0.250000	0.0400 (2)	
S1	0.71081 (4)	0.6684 (2)	0.07122 (12)	0.0889 (4)	
S2	0.66087 (3)	0.6590 (3)	0.12475 (12)	0.0818 (4)	0.92
S2A	0.6778 (4)	0.542 (3)	0.1952 (12)	0.0818 (4)	0.08
O1	0.53986 (6)	0.4747 (4)	0.28943 (18)	0.0481 (5)	
O1W	0.46681 (7)	−0.0863 (4)	0.32936 (19)	0.0521 (6)	
H1WA	0.464697	−0.113169	0.404242	0.078*	
H1WB	0.452668	−0.186774	0.293346	0.078*	
O2	0.52498 (6)	0.2365 (4)	0.43551 (19)	0.0517 (6)	
C1	0.72057 (15)	0.9590 (11)	0.1424 (5)	0.0997 (17)	
H1A	0.744854	0.960903	0.172016	0.120*	
H1B	0.717162	1.090218	0.083028	0.120*	
C3	0.67286 (13)	0.8089 (10)	0.2663 (5)	0.0857 (15)	
H3A	0.685568	0.684922	0.316278	0.103*	0.92
H3B	0.685947	0.751848	0.339876	0.103*	0.08
C4	0.64166 (12)	0.8763 (9)	0.3330 (4)	0.0795 (13)	
H4A	0.628523	0.999583	0.285669	0.095*	
H4B	0.649753	0.953975	0.408622	0.095*	
C5	0.61713 (10)	0.6719 (7)	0.3618 (4)	0.0603 (9)	
H5A	0.630382	0.538050	0.400644	0.072*	
H5B	0.606141	0.608592	0.286739	0.072*	
C6	0.58918 (10)	0.7526 (8)	0.4432 (3)	0.0599 (9)	
H6A	0.600257	0.838442	0.512319	0.072*	
H6B	0.574126	0.870293	0.399280	0.072*	
C7	0.56689 (10)	0.5489 (8)	0.4891 (3)	0.0601 (9)	
H7A	0.582235	0.427313	0.528863	0.072*	
H7B	0.552818	0.618412	0.550521	0.072*	
C8	0.54298 (8)	0.4159 (6)	0.3990 (3)	0.0420 (7)	
C2	0.69827 (16)	1.0064 (13)	0.2446 (6)	0.115 (2)	
H2A	0.713185	1.029186	0.317755	0.138*	
H2B	0.685666	1.159689	0.229112	0.138*	

### Diaquabis[5-(1,2-dithiolan-3-yl)pentanoato-κ2O,O']manganese(II) Atomic displacement parameters (Å^2^)

**Table:**

	*U*^11^	*U*^22^	*U*^33^	*U*^12^	*U*^13^	*U*^23^
Mn1	0.0538 (4)	0.0384 (4)	0.0279 (4)	0.000	0.0040 (3)	0.000
S1	0.0862 (8)	0.0991 (9)	0.0848 (8)	−0.0011 (6)	0.0323 (7)	−0.0086 (6)
S2	0.0697 (8)	0.1122 (11)	0.0638 (7)	−0.0159 (6)	0.0066 (6)	−0.0201 (6)
S2A	0.0697 (8)	0.1122 (11)	0.0638 (7)	−0.0159 (6)	0.0066 (6)	−0.0201 (6)
O1	0.0599 (13)	0.0495 (12)	0.0347 (11)	−0.0065 (10)	0.0005 (9)	0.0038 (9)
O1W	0.0740 (16)	0.0491 (12)	0.0338 (11)	−0.0126 (11)	0.0100 (10)	−0.0009 (10)
O2	0.0670 (15)	0.0548 (12)	0.0334 (11)	−0.0132 (11)	0.0037 (10)	0.0017 (10)
C1	0.087 (3)	0.102 (4)	0.116 (4)	−0.021 (3)	0.044 (3)	−0.012 (3)
C3	0.077 (3)	0.113 (4)	0.069 (3)	−0.030 (3)	0.020 (2)	−0.022 (3)
C4	0.070 (3)	0.087 (3)	0.084 (3)	−0.018 (2)	0.027 (2)	−0.023 (2)
C5	0.055 (2)	0.072 (2)	0.055 (2)	−0.0052 (17)	0.0076 (16)	−0.0035 (17)
C6	0.058 (2)	0.071 (2)	0.052 (2)	−0.0103 (18)	0.0088 (16)	−0.0134 (18)
C7	0.063 (2)	0.080 (2)	0.0375 (17)	−0.0215 (19)	0.0073 (15)	−0.0100 (17)
C8	0.0471 (16)	0.0460 (16)	0.0334 (15)	0.0020 (12)	0.0066 (12)	−0.0007 (12)
C2	0.095 (4)	0.128 (5)	0.129 (5)	−0.052 (4)	0.054 (3)	−0.048 (4)

### Diaquabis[5-(1,2-dithiolan-3-yl)pentanoato-κ2O,O']manganese(II) Geometric parameters (Å, º)

**Table:**

Mn1—O1	2.258 (2)	C3—H3A	0.9800
Mn1—O1^i^	2.258 (2)	C3—H3B	0.9800
Mn1—O1W^i^	2.125 (2)	C3—C4	1.492 (6)
Mn1—O1W	2.125 (2)	C3—C2	1.477 (7)
Mn1—O2^i^	2.237 (2)	C4—H4A	0.9700
Mn1—O2	2.237 (2)	C4—H4B	0.9700
S1—S2	2.0443 (18)	C4—C5	1.500 (6)
S1—S2A	2.042 (12)	C5—H5A	0.9700
S1—C1	1.787 (6)	C5—H5B	0.9700
S2—C3	1.797 (5)	C5—C6	1.509 (5)
S2A—C3	1.659 (14)	C6—H6A	0.9700
O1—C8	1.251 (4)	C6—H6B	0.9700
O1W—H1WA	0.8501	C6—C7	1.504 (5)
O1W—H1WB	0.8502	C7—H7A	0.9700
O2—C8	1.272 (4)	C7—H7B	0.9700
C1—H1A	0.9700	C7—C8	1.498 (5)
C1—H1B	0.9700	C2—H2A	0.9700
C1—C2	1.483 (7)	C2—H2B	0.9700
			
O1^i^—Mn1—O1	87.84 (12)	C2—C3—S2A	117.1 (6)
O1W—Mn1—O1	144.53 (8)	C2—C3—H3A	106.0
O1W^i^—Mn1—O1^i^	144.53 (8)	C2—C3—H3B	93.0
O1W—Mn1—O1^i^	98.02 (9)	C2—C3—C4	117.6 (5)
O1W^i^—Mn1—O1	98.01 (9)	C3—C4—H4A	108.0
O1W—Mn1—O1W^i^	97.07 (13)	C3—C4—H4B	108.0
O1W—Mn1—O2	87.45 (8)	C3—C4—C5	117.4 (4)
O1W—Mn1—O2^i^	104.17 (9)	H4A—C4—H4B	107.2
O1W^i^—Mn1—O2	104.17 (9)	C5—C4—H4A	108.0
O1W^i^—Mn1—O2^i^	87.45 (8)	C5—C4—H4B	108.0
O2—Mn1—O1	57.76 (8)	C4—C5—H5A	108.9
O2^i^—Mn1—O1^i^	57.76 (8)	C4—C5—H5B	108.9
O2—Mn1—O1^i^	108.39 (9)	C4—C5—C6	113.2 (3)
O2^i^—Mn1—O1	108.39 (9)	H5A—C5—H5B	107.8
O2^i^—Mn1—O2	162.59 (13)	C6—C5—H5A	108.9
C1—S1—S2	93.99 (17)	C6—C5—H5B	108.9
C1—S1—S2A	96.9 (4)	C5—C6—H6A	108.4
C3—S2—S1	92.66 (16)	C5—C6—H6B	108.4
C3—S2A—S1	97.0 (7)	H6A—C6—H6B	107.5
C8—O1—Mn1	91.54 (19)	C7—C6—C5	115.6 (3)
Mn1—O1W—H1WA	127.7	C7—C6—H6A	108.4
Mn1—O1W—H1WB	127.8	C7—C6—H6B	108.4
H1WA—O1W—H1WB	104.5	C6—C7—H7A	107.9
C8—O2—Mn1	91.98 (18)	C6—C7—H7B	107.9
S1—C1—H1A	109.2	H7A—C7—H7B	107.2
S1—C1—H1B	109.2	C8—C7—C6	117.8 (3)
H1A—C1—H1B	107.9	C8—C7—H7A	107.9
C2—C1—S1	111.9 (4)	C8—C7—H7B	107.9
C2—C1—H1A	109.2	O1—C8—O2	118.7 (3)
C2—C1—H1B	109.2	O1—C8—C7	122.7 (3)
S2—C3—H3A	106.0	O2—C8—C7	118.5 (3)
S2A—C3—H3B	93.0	C1—C2—H2A	108.6
C4—C3—S2	111.8 (3)	C1—C2—H2B	108.6
C4—C3—S2A	124.5 (6)	C3—C2—C1	114.7 (5)
C4—C3—H3A	106.0	C3—C2—H2A	108.6
C4—C3—H3B	93.0	C3—C2—H2B	108.6
C2—C3—S2	108.7 (4)	H2A—C2—H2B	107.6
			
Mn1—O1—C8—O2	0.3 (3)	S2—C3—C2—C1	−29.7 (8)
Mn1—O1—C8—C7	−179.4 (3)	S2A—S1—C1—C2	−8.4 (7)
Mn1—O2—C8—O1	−0.3 (3)	S2A—C3—C4—C5	14.5 (10)
Mn1—O2—C8—C7	179.4 (3)	S2A—C3—C2—C1	12.2 (11)
S1—S2—C3—C4	171.7 (4)	C3—C4—C5—C6	172.4 (4)
S1—S2—C3—C2	40.2 (5)	C4—C3—C2—C1	−157.9 (6)
S1—S2A—C3—C4	153.7 (5)	C4—C5—C6—C7	−171.9 (4)
S1—S2A—C3—C2	−15.7 (10)	C5—C6—C7—C8	−67.3 (5)
S1—C1—C2—C3	−0.3 (8)	C6—C7—C8—O1	−3.2 (5)
S2—S1—C1—C2	24.9 (5)	C6—C7—C8—O2	177.2 (3)
S2—C3—C4—C5	57.0 (6)	C2—C3—C4—C5	−176.2 (5)

Symmetry code: (i) −*x*+1, *y*, −*z*+1/2.

### Diaquabis[5-(1,2-dithiolan-3-yl)pentanoato-κ2O,O']manganese(II) Hydrogen-bond geometry (Å, º)

**Table:**

*D*—H···*A*	*D*—H	H···*A*	*D*···*A*	*D*—H···*A*
O1*W*—H1*WA*···O2^ii^	0.85	1.91	2.725 (3)	159
O1*W*—H1*WB*···O1^iii^	0.85	2.07	2.718 (3)	132
C2—H2*B*···S2*A*^iv^	0.97	2.12	3.043 (18)	158

Symmetry codes: (ii) −*x*+1, −*y*, −*z*+1; (iii) −*x*+1, *y*−1, −*z*+1/2; (iv) *x*, *y*+1, *z*.
